# The activity of miltefosine combined with oral probiotics in a mouse model of cutaneous leishmaniasis

**DOI:** 10.1590/0074-02760250056

**Published:** 2025-11-14

**Authors:** Yevva Cranshoff, Raquel Azevedo, Marcos Meuser Batista, Kelly Cristina Demarque, Roberson Donola Girão, Amanda Faier-Pereira, Beatriz Iandra da Silva Ferreira, Otacilio Moreira, Cynthia Machado Cascabulho, Ludmila Ferreira Fiuza, Guy Caljon, Maria de Nazaré Correia Soeiro

**Affiliations:** 1 Fundação Oswaldo Cruz-Fiocruz, Instituto Oswaldo Cruz, Laboratório de Biologia Celular, Rio de Janeiro, RJ, Brasil; 2 Fundação Oswaldo Cruz-Fiocruz, Instituto Oswaldo Cruz, Laboratório de Virologia e Parasitologia Molecular, Rio de Janeiro, RJ, Brasil; 3 Fundação Oswaldo Cruz-Fiocruz, Instituto Oswaldo Cruz, Plataforma de Citometria, Rio de Janeiro, RJ, Brasil; 4 University of Antwerp, Laboratory of Microbiology, Parasitology and Hygiene, Wilrijk, Belgium

**Keywords:** Leishmania amazonensis, in vivo, probiotics, antileishmanials, combo treatment

## Abstract

**BACKGROUND:**

Leishmaniasis, caused by the protozoan *Leishmania*, is a neglected tropical disease (NTD) with diverse clinical forms, the most common being cutaneous leishmaniasis (CL). Antileishmanial treatments rely on a small arsenal of chemoherapeutic agents, which are outdated, toxic, and increasingly ineffective due to drug resistance. New antileishmanial treatments and/or adjunctive therapies are warranted.

**OBJECTIVES:**

Given the role of microbiota in modulating host immunity, we explored whether probiotics (PB8-multistrain probiotic blend, or *Lactobacillus rhamnosus* GG, LGG-single strain) alone or in combination with the reference drug miltefosine (ML) could improve clinical outcomes against *Leishmania amazonensis* infection in a BALB/c mouse model for CL.

**METHODS:**

Mice were administered probiotics [gavage, 10^9^ colony-forming units (CFU)] for a 7-day pre-treatment before infection, followed by another 14-day probiotic treatment with ML co-administration. Paw lesions were measured using a digital calliper, and parasite loads were determined through lesion imprinting and quantitative polymerase chain reaction (qPCR). The potential immunoregulatory effects of probiotic administration on the mouse serum cytokine profiles were investigated via flow cytometry.

**FINDINGS AND MAIN CONCLUSIONS:**

Probiotics alone reduced lesion size slightly, with PB8 achieving a 32% and LGG a 10% reduction at the endpoint (47-50 days post-infection, dpi). The combination of PB8 with a suboptimal ML dose (4 mg/kg/day) reduced the lesion size by 74% compared to the vehicle-treated mice, while ML alone achieved 53%. These findings were corroborated by amastigote quantification via imprinting (light microscopy) and qPCR: PB8 plus ML reduced parasite load by 76% and 87%, respectively. Multiplex cytokine analysis [interferon (IFN)-γ, interleukin (IL)-6, IL-10, IL-12p70, tumour necrosis factor (TNF) and chemokine CCL2] showed reduced serum CCL2 in PB8-cotreated groups. This suggests that PB8 could modulate serum cytokine levels to mitigate the risk of excessive inflammation, as elevated CCL2 is linked to disease exacerbation through monocyte recruitment. Our findings demonstrate the potential effect of probiotic administration to enhance antileishmanial efficacy of antiparasitic drugs.

Leishmaniasis, a neglected tropical disease (NTD), is caused by intracellular kinetoplastid protozoan parasites of the genus *Leishmania*, which belong to the Trypanosomatidae family.^1^ ^,^ ^2^ Ranked as the third most significant vector-borne disease worldwide by the World Health Organization (WHO), it is transmitted through the bites of infected female sand flies of the *Lutzomyia* (New World) or Phlebotomus (Old World) genera (Diptera: Psychodidae).^1^ ^,^ ^3^ Over 20 species of *Leishmania* can cause human leishmaniasis, leading to diverse and often unpredictable clinical manifestations.^1^ ^,^ ^4^ Leishmaniasis is classified into three primary clinical forms: visceral (VL), mucocutaneous (MCL), and cutaneous (CL). While VL is the most fatal, CL is the most prevalent, characterised by non-lethal skin lesions.^1^

Cutaneous leishmaniasis is endemic in over 90 countries, with up to 95% of cases concentrated in South America (including Brazil), the Mediterranean region, Asia, and the Middle East.^5^ The global incidence of CL ranges from 600,000 to 1 million new cases annually, with approximately 60,000 deaths.^1^ ^,^ ^6^

Each case of CL can leave patients with permanent scars, disabilities, and stigmas, which significantly impact on their mental health and social relationships. These consequences pose a substantial public health burden, particularly in impoverished regions where risk factors such as poverty and malnutrition prevail.^7^ ^,^ ^8^ CL typically develops weeks to months after the initial sand fly bite, presenting as single or multiple skin lesions that may progress into severe chronic ulcers. Although CL lesions are often self-healing, resolution can take years and may result in significant scar tissue formation and secondary infections.^9^ In more extreme cases, disseminated and mucosal lesions may develop, causing disfigurement and increasing morbidity.^1^ ^,^ ^9^ Also, infections caused by certain *Leishmania* species, such as *L. aethiopica*, *L. amazonensis*, and *L. braziliensis*, can progress from cutaneous to mucocutaneous symptoms, referred to as tegumentary leishmaniasis. These cases may include destructive nasopharyngeal tissue involvement affecting the mouth, nose, throat, and adjoining structures.^10^ ^,^ ^11^ The likelihood and severity of clinical manifestations are influenced by human genetic predisposition, immunocompromised conditions [*e.g.*, human immunodeficiency virus (HIV)], parasite or sand fly genetic diversity, and environmental conditions such as climate change.^12^ ^,^ ^13^

Currently, no antileishmanial human vaccines are anticipated in the foreseeable future.^14^ In addition, CL treatment is based on a small therapeutic arsenal (Table), of which most drugs face emerging parasite drug resistance and high failure rates in endemic countries, often due to inappropriate dosing and treatment regimens.^15^ The available treatment options compromise the first-line chemotherapy drugs pentavalent antimonial (Glucantime^®^ and Pentostam^®^), and the second-line chemotherapy drugs (liposomal) amphotericin B [(L-)AmB; Ambisome^®^], pentamidine isethionate (Pentam^®^), miltefosine (ML) (Milteforan™), and paromomycin. ML was a major therapeutic advance, the first oral drug in the therapeutic arsenal against leishmaniasis.^15^ ^,^ ^16^ The drug’s effectiveness depends heavily on the *Leishmania* species and the endemic region.^15^ ^,^ ^16^ ^,^ ^17^ In addition to emerging drug resistance, treatments for leishmaniasis face challenges such as safety and efficacy concerns when used as monotherapies, lengthy courses, limited administration routes (parenteral only, except Milteforan), high costs, cold storage needs, and toxicity that impacts patient compliance and requires monitoring in specialised centres.^18^

**TABLE:** Conventional chemotherapeutic drugs against human leishmaniasis. The table outlines conventional treatment options for human leishmaniasis, detailing the administration route, adverse effects, key advantages, and notable drawbacks

Chemotherapeutic drugs for human leishmaniasis^*^				
	Routes of administration	Adverse effects	Advantages	Drawbacks
Pentavalent Antimonial	Intramuscular; intravenous & intralesional	Cardiotoxicity, nephrotoxicity & hepatotoxicity	Lower costs & readily available in endemic areas	Prolonged treatment regime, painful injections & reported drug resistance
Amphotericin B	Intralesional	Nephrotoxicity, hypokalaemia & ISRs (*i.e.* irritation, swelling, redness)	Lower parasite drug resistance	Hospitalisation is required for injection
Liposomal Amphotericin B	Intravenous	Mild nephrotoxicity, hepatotoxicity & teratogenicity	High efficacy & reduced toxicity	High costs & poor patient compliance
Miltefosine (Milteforan)	Oral	Nephrotoxicity, hepatotoxicity & teratogenicity	High efficacy	High costs
Paramomycin	Iintramuscular (visceral form) and topical (cutaneous form)	Nephrotoxicity & hepatotoxicity	High efficacy & low costs	Efficacy variability due to the geographical area
Pentamidine	IM	Cardiotoxicity & hepatotoxicity	Short-term treatment regimen	Efficacy depends on *Leishmania* species

*Adapted by Pradhan et al.^52^; IM: intramuscular; ISRs: injection site reactions.

Recent advances in metagenomics and high-throughput DNA sequencing have revolutionised our understanding of the microbiome’s dynamic influence on human health and disease.^19^ ^,^ ^20^ Consequently, host-microbiota interactions have emerged as a key focus for developing therapeutic and preventive strategies in public health. Current literature highlights the role of microbiota in disease outcomes and describes how dysbiosis contributes to chronic illness progression by influencing host physiology and immunity.^21^ The term ‘microbiota’ refers to the collective, dynamic population of microorganisms associated with vertebrate and invertebrate species.^22^ ^,^ ^23^ These microorganisms include commensal, symbiotic, and pathogenic species, whose composition is shaped by site-specific factors and the indigenous microbial community.^24^ Common classifications include regions such as the gut, skin, oral cavity, respiratory tract, and vagina, with bacterial diversity varying based on the host’s size, age, and gender.^24^ ^,^ ^25^ Over evolutionary time, the microbiota and host have co-evolved to form symbiotic relationships that contribute to physiological processes such as metabolism, barrier function, immune responses, and haematopoiesis.^26^ ^,^ ^27^ This symbiosis plays a central role in maintaining homeostasis, while competitive microbial interactions and immune regulation help prevent colonisation by harmful pathobionts - also known as colonisation resistance.^28^ ^,^ ^29^ ^,^ ^30^

Various factors, including aging, dietary changes, antibiotic use, geographical shifts, host genetics, allergies, and diseases, can influence fluctuations in microbiota composition.^22^ ^,^ ^31^ Although these changes are not always detrimental, they can lead to dysbiosis, a microbial imbalance within specific body regions. Dysbiosis is characterised by reduced bacterial diversity, often resulting from the loss of indigenous microbial communities and the overgrowth of opportunistic pathogenic bacteria.^32^ This imbalance can disrupt essential bodily functions and contribute to the onset of a range of conditions, including respiratory illnesses, cancers, metabolic and autoimmune disorders, skin diseases, and infections.^24^ Therefore, targeted approaches to manipulate microbiota composition and functionality offer innovative strategies for enhancing host resilience, reducing pathogen colonisation, and fostering a balanced microbial ecosystem that supports immune defence against human pathogens. To illustrate, a study by Farias-Amorim et al. revealed that skin microbiota, particularly *Staphylococcus aureus*, influence disease outcomes in *Leishmania braziliensis* infections by modulating inflammatory responses through interleukin (IL)-1β.^33^ It highlights the microbiome’s role in delayed healing and suggests potential pathways for host-directed therapies to reduce inflammation and pathology in CL.

Among the various strategies explored in microbiota modulation, probiotics have garnered significant attention. Probiotics are viable, non-pathogenic symbiotic microorganisms that, when administered in sufficient quantities, deliver significant health benefits to the host, *i.e.*, restoring the microbiota balance in patients by stimulating the immune system, metabolism and antipathogenic activities.^34^ They are prescribed as dietary supplements for clinical interventions, such as gastro-intestinal disorders.^22^ ^,^ ^35^ The underlying work mechanisms of probiotics involve restoring microbial dysbiosis and maintaining the hosts’ microbial homeostasis by occupying the host tissues and preventing colonisation by acquired pathogens.^23^ ^,^ ^36^ Given the probiotics’ inherent biological nature, resistance is not anticipated to arise, unlike the emerging occurrence observed in conventional CL drug approaches and oral antibiotic use, of which the latter is often included in the treatment regimen of inflammatory cutaneous disorders.^21^

Primarily, *Lactobacillus*, *Saccharomyces*, and *Bifidobacterium* are described by literature as suitable probiotics, having an instrumental role in the modulation of the microbiota’s composition and its corresponding immunological and physiological activities.^37^ For example, *Lactobacillus casei*, *ATCC7469* strain, induces an early protective immune response against hemiparasite *Babesia microti* in mice.^38^ Other (sub-)species of *Lactobacilli* have been extensively studied in murine models, demonstrating their (immunobiotic) role in terms of protection against several parasitic diseases, including Toxoplasmosis, Trichinellosis, Giardiasis, and Malaria.^39^ ^,^ ^40^ ^,^ ^41^ ^,^ ^42^

In this sense, given the role of microbiota in modulating host physiology and immunity, we investigated whether probiotics (PB8-multistrain probiotic blend, or *Lactobacillus rhamnosus* GG, LGG-single strain) alone or in combination with the reference drug ML could improve outcomes against *L. amazonensis* experimental infection in a BALB/c mouse model for CL.

## MATERIALS AND METHODS

*Animals* - Male and female BALB/c mice weighing 18-21 g were provided by the Institute of Sciences and Technologies in Biomodels (ICTB-Fiocruz, Rio de Janeiro, Brazil). The animals were housed in polypropylene cages (maximum six animals per cage) at ambient temperature (22 ± 2ºC) under a 12 h light/dark cycle, supplying sterilised water and chow *ad libitum*.

*Parasites* - BALB/c mice were inoculated in the foot paws (subcutaneously) with 20 μL containing 5 × 10^5^ *Leishmania (L.) amazonensis* (MHOM/BR/77/LTB0016) amastigotes.^43^ Thirty days post-infection, skin lesions were routinely removed aseptically and mechanically dispersed by pipetting, from which the amastigotes were purified, as reported by Santos et al.^43^

*Oral probiotics* - The probiotics used include multi-strain probiotic blend PB8 (composed of *Lactobacillus acidophilus LA-14* ^®^, *Bifidobacterium lactis BL-04* ^®^, *Lactobacillus salivarius LS-33* ^®^, *Bifidobacterium bifidum BB-06™*, *Bifidobacterium longum BL-05™*, *Lactobacillus rhamnosus LGG* ^®^, *Lactobacillus casei LC-11* ^®^, from Nutrition Now), and single-strain probiotic *Lactobacillus rhamnosus GG* (DSM 33156) LGG (from Bifilac Geoflora). Both probiotics were administered orally (gavage) in a dose of 10^9^ colony-forming units (CFU) per 200 uL, as reported by Garfias et al.^44^ Daily, the probiotic suspensions were freshly prepared by diluting a single capsule in ultrapure and sterile water.

**Fig. 1:**
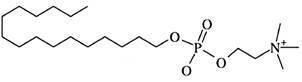
chemical structure of reference drug miltefosine (ML) (Adapted from Palic et al.).^16^

*Reference drug* - For *in vitro* studies, ML (Fig. 1) was prepared as a stock compound solution (30 mM) in dimethyl sulfoxide (DMSO), as reported by Santos et al.^45^ For *in vivo* studies, ML (Milteforan™, from Virbac) was prepared in ultrapure and sterile water before use and administered through oral gavage, as described by Lin et al., using the optimal dose of 40 mg/kg/day (alone, ML 40) or the suboptimal dose of 4 mg/kg/day (combined, ML 4) as a post-infection treatment from 15 to 19 dpi (short-term) and from 15 to 35 dpi (long-term).^17^

*In vivo activity studies* - *In vivo*, probiotic mono- and combinatorial therapy with ML was assessed using a mouse model of *L. amazonensis* infection. Probiotic (10^9^ CFU of PB8 or LGG in 200 µL) were given to male BALB/c mice orally (via gavage) for seven consecutive days. Subsequently, animals were subcutaneously infected in the right hind paw with 5 x 10^5^ *L. amazonensis* amastigotes purified from animal lesions. The vehicle group received 200 µL of ultrapure sterile water via gavage. Following infection, the probiotic and vehicle treatment were continued for another fourteen days, resulting in twenty-one days of probiotic or vehicle administration. Each group consisted of five to six mice, with PB8 monotherapy also evaluated in female BALB/c mice (n = 5). ML 4 was administered or not over a short-term regimen of five consecutive days (15 to 19 days post-infection, dpi) in vehicle - and probiotic-treated groups. The combination of probiotics with ML 4 was also further evaluated in a long-term regimen over twenty-one consecutive days (15 to 35 dpi). Control groups included ML alone at both optimal (ML 40) and suboptimal (ML 4) doses.

*Treatment evaluation* - Throughout the assays, the paw lesion growth in mice was measured thrice weekly in three dimensions (height, width, and depth, mm³) using a digital calliper. Upon reaching the endpoint (47-50 dpi), mice were euthanised by isoflurane overdose (Bonther Equipamentos - São Paulo - Brazil).^46^ The experimental endpoint included collecting the infected mouse hind paws (or lesions) and a cardiac blood sample (1 mL). The amastigote load in the lesions and the mouse inflammatory profiles were assessed using histopathology and lesion imprint analyses. quantitative polymerase chain reaction (qPCR) analyses were performed at the endpoint on the collected lesions to determine the parasite tissue burden. The serum isolated from the obtained blood samples was used for cytokine profiling with the Cytometric Bead Array (CBA) Mouse Inflammation Kit.

*Quantification of tissue parasite burden by qPCR* - The samples were homogenised using the Tissue rupture II (from Qiagen, Hilden, Germany) in 200 µL of the Tissue Lysis Buffer from the High Pure PCR Template Preparation kit (Roche, Basel, Switzerland). After homogenisation, DNA was isolated using the High Pure PCR Template Preparation kit according to the manufacturer’s instructions. At the final step of the protocol, DNA was eluted in 100 μL and stored at -20ºC until use. The qPCR assays were performed using the TaqMan system with primers and probes designed for the 18S rDNA target in *Leishmania*.^47^ A TaqMan assay for the mouse GAPDH gene was used as an internal control to normalise the parasitic load (from Applied Biosystems, Foster City, USA, Cat. No.: Cat. Nº 4352339E). Reactions were conducted using 10 µL of the FastStart Universal Master Mix [2×] (from Roche, Basel, Switzerland) 150 nM primer 18S rDNA F (5’-GTACTGGGGCGTCAGAGGT-3’), 300 nM primer 18S rDNA R (5’-TGGGTGTCATCGTTTGCAG-3’), 200 nM 18S rDNA Tq (5’- FAM-AATTCTTAGACCGCACCAAG-NFQ-MGB-3’), 1 μL Mouse GAPDH Endogenous Control [20×] (VIC/NFQ/MGB, Applied Biosystems, Foster City, California, USA) and 5 μL DNA in a final volume of 20 μL. Cycling conditions include a first step at 95ºC for 5 min, followed by 40 cycles at 94ºC for 15 s and at 60ºC for 1 min. The amplifications were performed in a Quantstudio 3 Real-Time PCR system (from Applied Biosystems, Foster City, California, USA). For the standard curve preparation to the absolute quantification by qPCR, negative samples (50 mg) were spiked with 1 × 10^6^ *L. amazonensis* amastigotes before DNA extraction. Standard curves were prepared by DNA serial dilutions in a 1:10 dilution factor and used in each qPCR plate, reaching from 1 × 10^6^ to 1 parasite equivalents/reaction and 50 to 5 × 10^-3^ mg of mouse tissue. The mouse tissue loads were used to normalise the parasitic loads, expressed as Equivalents of parasite/mg of mouse tissue. All experiments included positive and negative controls. In each batch of DNA extraction, one tube containing 200 μL molecular biology water was used as a negative control. In addition, two wells containing 5 μL of ultrapure water were used as negative template control. As positive controls, 5 μL each of *L. amazonensis* DNA (at 1 pg/mL and 100 fg/μL) were used. Each sample was assayed in technical duplicates. A sample was considered positive (detectable *Leishmania* DNA) when the amplification curve for the *Leishmania* target exceeded the fluorescence threshold (set at 0.02) during the 40 PCR cycles, resulting in a cycle threshold (Ct) value. A sample was considered negative (non-detectable *Leishmania* DNA) when the amplification curve did not exceed the threshold within 40 cycles.

*Cytometric beads array analysis by flow cytometry* - At the endpoint, mouse blood samples (approximately 1 mL) were collected through cardiac puncture.^48^ The blood samples were centrifuged at 2,000×g for 20 min at 4ºC, and the serum was isolated and stored at -80ºC until use. Later, the sera was assessed using the Mouse inflammatory cytokine CBA kit (BD Biosciences, San Diego, USA) for the serum level quantification (pg/mL) of six specific mouse cytokines, including interferon-gamma (IFN-γ), interleukin (IL)-6, IL-10, IL-12p70, tumour necrosis factor (TNF) and chemokine CCL2 (also known as Monocyte Chemoattraction Protein-1, MCP-1) as reported by Almeida et al.^49^ The cytokine capture bead, PE detection reagent, and recombinant standards or test samples were incubated for 3 h at room temperature according to the manufacturer’s instructions.^50^ The FACSCanto flow cytometer (BD Biosciences, San Diego, USA) was used to acquire the data and FCAP Array™ software served to generate results in graphical format.

*Lesion imprint analysis by light microscopy* - Following the qPCR preparation, the remaining (or distal) part of the mouse lesions was used to prepare the imprints, which were fixed with methanol and stained by Giemsa.^43^ The stained imprints were used to evaluate the inflammatory profile qualitatively - including cellularity (absence or presence) and the type of inflammation (*e.g.*, pyogranulomatous) - and tissue alterations, such as tissue and cell debris. Additionally, the cell profiles, including macrophages, neutrophils, lymphocytes, and *L. amazonensis* amastigotes, were quantified using a light microscope at 1000× magnification across 10-20 random fields of view (FOV).^43^

*Histopathological analysis by light microscopy* - At the endpoint, mice paw lesions were collected, the crusts removed, and a transverse cut made in the region of the direct plantar pad, where the most proximal area was dissected and sent for qPCR analysis and distal portion processed for histopathological examination. The samples were fixed in 10% buffered formalin in phosphate-buffered saline (Sigma-Aldrich, St. Louis, MO, United States), and the material was then cleaved lengthways so that the sections could show all the layers of the tegument. All samples were processed using paraffin-embedding techniques, sectioned at 4 µm thickness and stained using Haematoxylin-Eosin (Sigma-Aldrich, St. Louis, MO, United States). Histopathological examination was conducted to evaluate the cell profiles (*e.g.*, neutrophilic or lymphocytic infiltrate), the inflammatory profile (*e.g.*, granulomatous or pyogranulomatous), and tissue alterations in the epidermis (*e.g.*, erosion/ulcers, intra- or intercellular oedema lesion, hyperkeratosis, and hyperplasia) and dermis (*e.g.*, inflammation, necrosis, and preservation of superficial dermis). Additionally, a semi-quantitative assessment of amastigote forms at the inner and outer edges was performed using light microscopy in 20 random FOVs at 1,000× magnification.^42^ The diagnosis was based on the International Harmonisation of Nomenclature and Diagnostic Criteria for Lesions in Rats and Mice.^51^

*Activity against axenic amastigotes (ex vivo assay)* - *Ex vivo* assays were performed to evaluate the direct effect of reference drug ML (Milteforan™), and probiotics PB8 and LGG, on the free *L. amazonensis* amastigotes purified directly from mouse paw lesions as described by Santos et al.^43^ The assays (1 × 10^6^ parasites/mL in 96-well microplates, 5% CO_2_ incubator) were performed at 32ºC by adding increasing concentrations of the studied agents and incubating in RPMI 1640 medium (pH 7.2 to 7.4) without phenol red (Gibco BRL) supplemented with 5% foetal bovine serum (FBS), 1% L-glutamine, 1% penicillin-streptomycin. After 48 h of incubation, the parasite viability was evaluated by light microscopy (at 400× magnification) using Neubauer counting chambers for the oral probiotics (including untreated-and miltefosine-treated controls) and by AlamarBlue^TM^ (resazurin sodium salt from Invitrogen) colorimetric assay at 560-590 nm for the reference drug. The half-maximal inhibitory concentration (IC_50_) of each test agent was calculated - representing the minimum compound concentration capable of reducing the parasite load by 50%, respectively, by non-linear regression analysis using GraphPad Prism v.9.3.0. The assays were run thrice in duplicate.

*Statistical analysis* - For *ex vivo*, the statistical analysis was performed using a Student’s *t*-test with a significance level of p ≤ 0.05 [95% confidence interval (CI)] in GraphPad Prism v.9.3.0. Two independent replicates were analysed each time for the *ex vivo* activity assays. *In vivo* statistical analyses were performed using GraphPad Prism v9.3.0. For comparisons between two independent groups, an unpaired Student’s *t*-test was used with a significance level of p ≤ 0.05. For comparisons among three or more independent groups, an ordinary one-way analysis of variance (ANOVA) was applied, followed by Tukey’s multiple comparisons test, also with a significance threshold of p ≤ 0.05. Significant differences are indicated using asterisk: standard asterisk(s) (*) denote significance relative to the vehicle group, while underlined asterisk(s) (*/) indicate significant differences between experimental groups, respectively. The levels of significance are defined as followed: *≤ 0.05, **≤ 0.01, ***≤ 0.001. All *in vivo* analyses were performed with five to six mice per cage, per gender.

*Ethical statement* - All procedures were carried out following the Biosafety Guidelines in compliance with the Oswaldo Cruz Foundation (Fiocruz), and all animal procedures were performed in compliance with the Ethical Guidelines established and approved by the Committee of Ethics for the Use of Animals (CEUA) of Fiocruz (Licence number: L-038/2017-A4, L-017/2023). 

## RESULTS

**Fig. 2:**
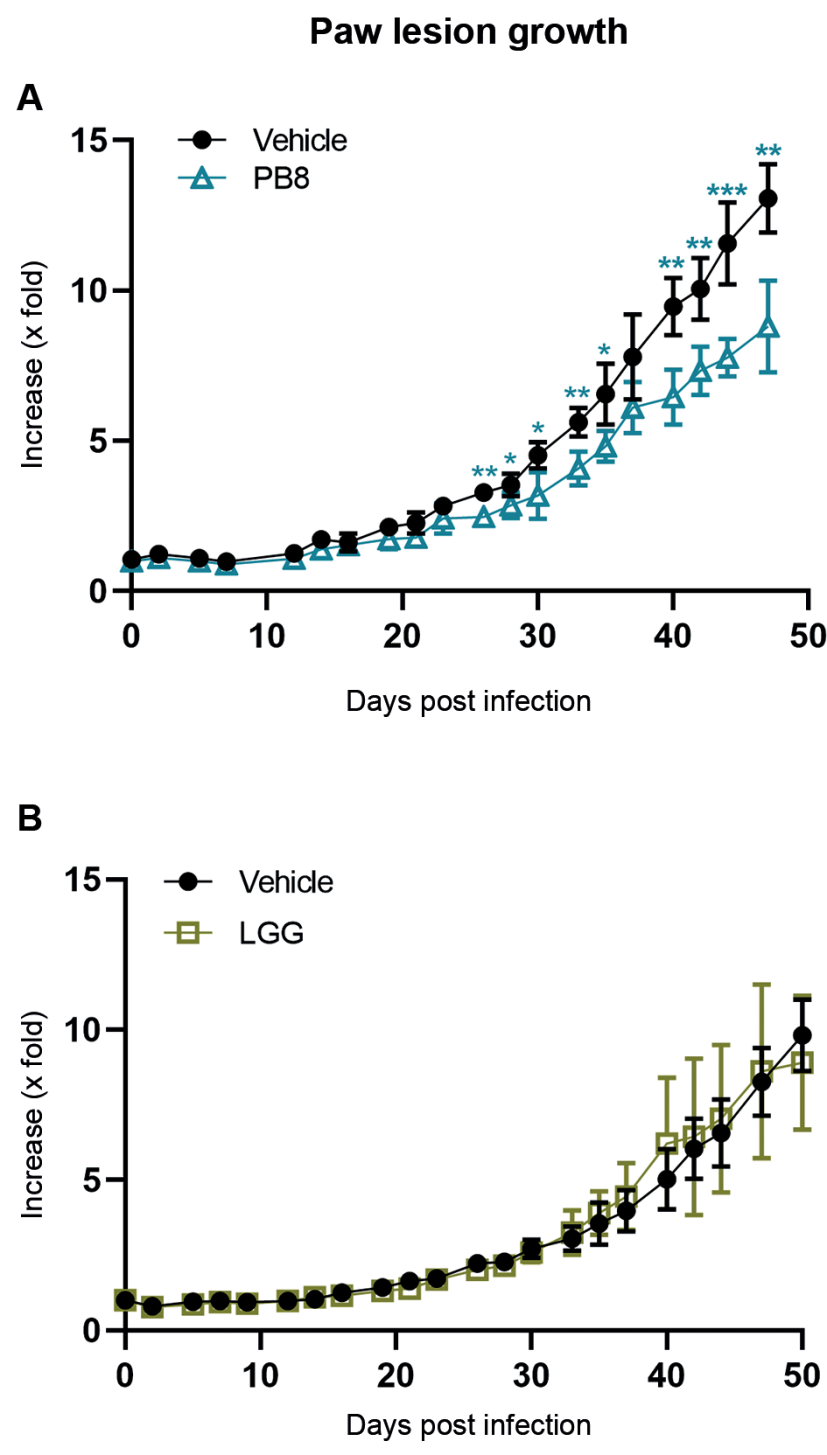
the impact of probiotics in paw lesion increase (expressed in x-fold). Male BALB/c mice were experimentally infected with *Leishmania amazonensis* amastigotes and treated with multi-strain probiotic blend PB8 (Fig. 2A) or *Lactobacillus rhamnosus* GG (LGG) (Fig. 2B). GraphPad Prism v9.3.0 was used to perform the statistical analysis (unpaired student t-test) and graph design. Statistical differences relative to the vehicle are indicated by asterisk(s). Levels of significance are denoted as followed: * ≤ 0.05, **≤ 0.01 and ***≤ 0.001.

*In vivo activity studies* - PB8 or LGG administrated in male BALB/c mice reduced paw lesion sizes by up to 32% and 10%, respectively, at 50 dpi (endpoint), compared to the vehicle-treated group and only the former induced a statistically significant reduction compared to the vehicle (p = 0.0024) (Fig. 2).

**Fig. 3:**
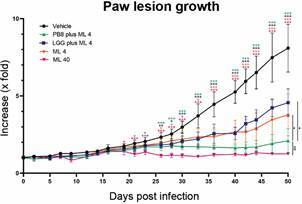
the impact of probiotics co-administration with Milteforan (ML) on the paw lesion size (expressed in x-fold increase). Male BALB/c mice were experimentally infected with *L. amazonensis* amastigotes. The graph demonstrates the lesion growth in groups co-treated with multi-strain probiotic blend PB8 or *Lactobacillus rhamnosus* GG (LGG) and a suboptimal dose of ML [ML 4, administered from 15 to 35 days post-infection (dpi) ], as well as in control groups (vehicle and ML 40, administered from 15 to 19 dpi). Graph design and statistical analysis [one-way analysis of variance (ANOVA) followed by Tukey’s multiple comparisons test] were performed using GraphPad Prism v9.3.0. Statistical differences relative to the vehicle are indicated by asterisk(s), while underlined asterisks indicate significant differences relative to the PB8+ML4 group (at 50 dpi). Levels of significance are denoted as followed: *≤ 0.05, **≤ 0.01 and ***≤ 0.001.

**Fig. 4:**
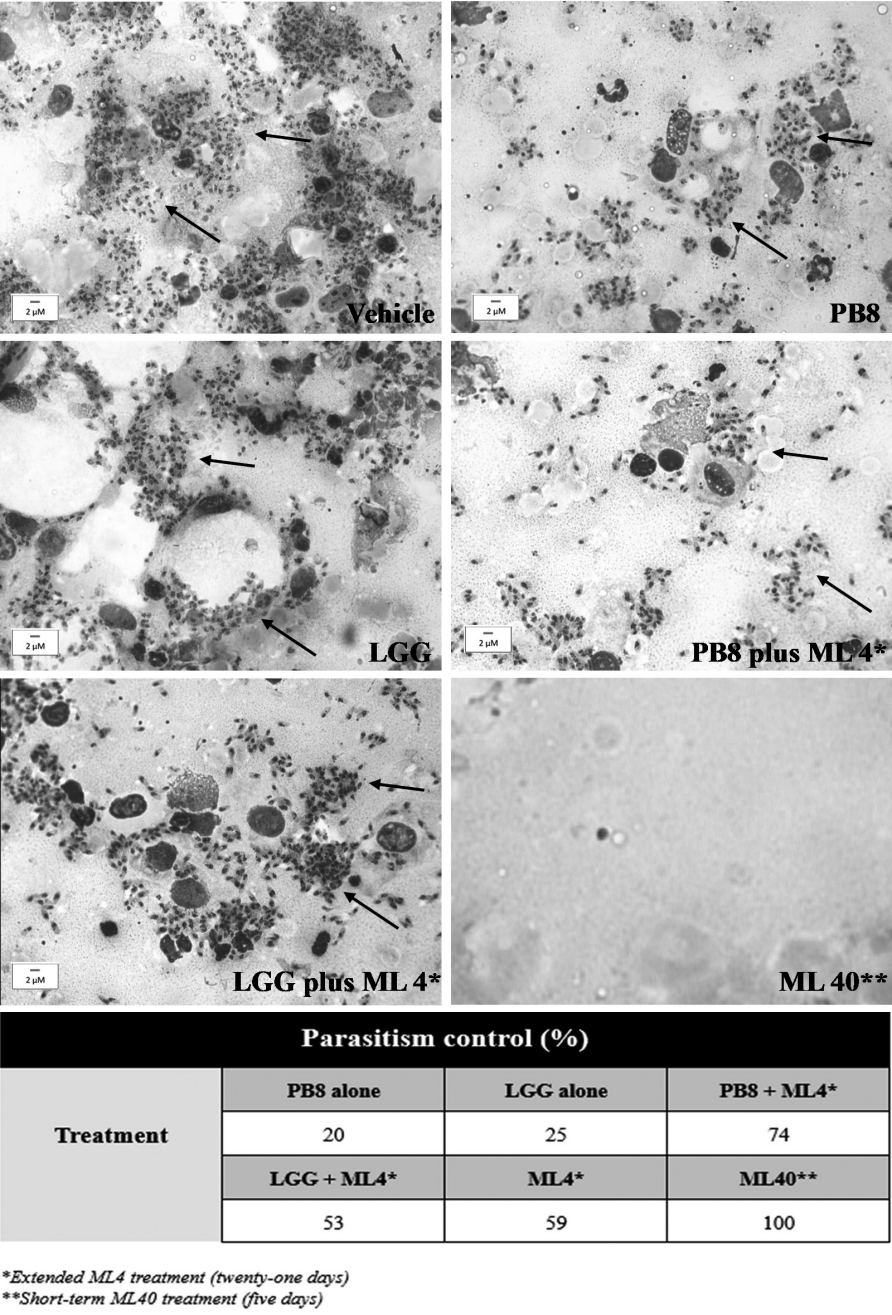
determination of the percentage in parasitism reduction in probiotics groups alone and in co-administration with Milteforan (ML) compared to vehicle-treated mice and the light microscopy analysis of parasite nests at the lesions’ paw of *L.amazonensis*-infected mice. The quantifications were conducted by light microscopy at 1000x magnification in 20-30 random fields of view (FOVs). The black arrows indicate parasite nests. *Extended ML 4 regime (25-days) and **short-term ML40 regime (5-days).

**Fig. 5:**
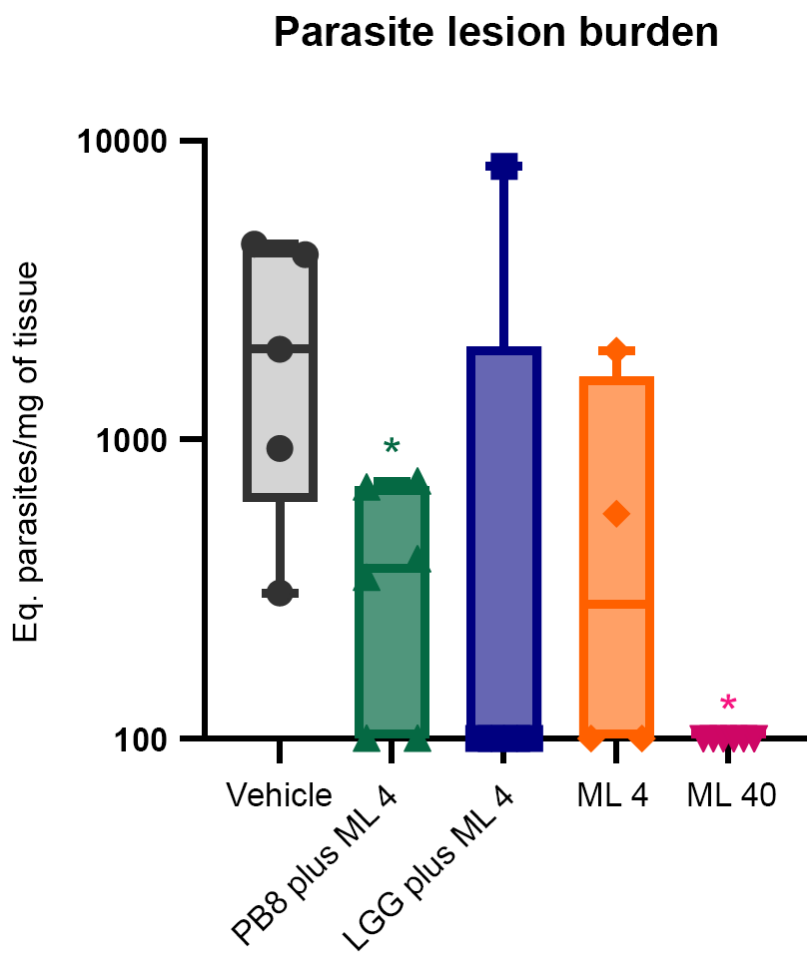
quantitative polymerase chain reaction (qPCR) analysis of parasite load in mice paw lesions. Male BALB/c mice were experimentally infected with *Leishmania amazonensis* amastigotes and subjected or not (vehicle) to probiotic administration [PB8 and *Lactobacillus rhamnosus* GG (LGG)] combined with miltefosine (ML, 4 mg/kg/day at long-term administration), and ML alone at suboptimal dose (4 mg/kg/day, long-term administration, ML 4). Also, ML at 40 mg/kg/day is displayed (given for five consecutive days as short-term administration, ML 40). The parasite loads are expressed as the equivalent (eq.) of parasites per mg of tissue. GraphPad Prism v9.3.0 was used to perform the statistical analysis (unpaired student t-test) and graph design. Statistical differences relative to the vehicle are indicated by asterisk(s). Levels of significance are denoted as followed: *≤ 0.05.

ML 40 therapy (15-19 dpi) achieved 95% of significant decline compared to the vehicle (p < 0.0001) (Fig. 3). Light microscopy of the lesion imprints confirmed parasitism reduction in the PB8 or LGG-treated mouse lesions, reaching up to 20% and 25%, respectively (Fig. 4). However, qPCR results did not show significant differences among the probiotic groups as compared to vehicle-treated mice (data not shown), except for ML 40 administration, which reached up to > 99% of parasite load decline (p = 0.01223) (Fig. 5).

A proof of concept, oral PB8 administration was also evaluated in female BALB/c mice in a single independent assay (data not shown). PB8-treated females presented smaller but non-significant lesions’ decrease compared to the vehicle-treated group.

Probiotics co-administered with ML (ML 4 for five days; short-term treatment) gave no enhanced in antiparasitic drug efficacy compared to the treatment with probiotics alone, reaching 15% and 17% of reduction in lesion size for PB8 and LGG respectively. These findings align with the imprint inspect, demonstrating 21% and 10% suppression in amastigote numbers, respectively for PB8 and LGG combined with a short-term treatment of ML. However, no statistical difference among the combos and vehicle groups was observed by the qPCR analysis (data not shown).

The most promising results were obtained when the ML (4 mg/kg/day) treatment regimen was extended from five to twenty-one days (15 to 35 dpi, prolonged) with probiotic administration (same regimen as previously described). Significantly, the PB8 plus ML 4 reduced the average mice lesion size by 74% compared to the vehicle group (p < 0.0001) (Fig. 3). ML 4 alone following the same long-term regimen, resulted in a significant but milder reduction in mouse lesion sizes (53%) at the endpoint (p = 0.0005) (Fig. 3). Once more, the LGG plus ML 4 combination approach proved less promising than its PB8 counterpart, achieving only a 44% reduction in lesion size, which was not significantly different as compared to the vehicle group at the endpoint (Fig. 3). At 50 dpi, no significant difference in lesion size reduction was observed between the PB8 plus ML 4 and the ML 40 groups (p = 0.6391). The imprint analysis by light microscopy again corroborated the higher parasite decline in the PB8 plus ML 4 group, reaching up to 74%, while combo LGG plus ML 4 and ML 4 alone, got maximum of 53% and 59% of parasitism decrease, respectively (Fig. 4). In imprints samples, while the number of amastigotes in vehicle-treated samples was about 340 amastigotes/field (20-random fields), and no parasites could be found in the imprints of the ML 40 group, even after exploring more than 50 random fields (Fig. 4). In addition, the qPCR analysis confirmed the statistically significant parasite suppression at the lesions induced by the PB8 plus ML 4, reaching 87% reduction, as compared to the vehicle-treated mice (p = 0.0282) (Fig. 5).

Besides the extended co-treatment with ML 4, we assessed the potential effects of prolonged probiotic (co-) administration by adding them directly in the drinking water but at this time, starting only after parasite infection (from 19 dpi to endpoint). Our data using male BALB/c mice did not show improved antiparasitic drug efficacy regarding the lesion measurements, lesion imprint and qPCR samples (data not shown).

Regarding the mouse inflammatory profile, a qualitative analysis on the lesion samples was performed by light microscopy assessing cellularity and tissue alterations in the epidermis (*e.g.*, erosion/ulcers, oedema, hyperkeratosis and hyperplasia). All probiotic (-co-) treated groups exhibited a pyogranulomatous profile, characterised by a predominance of neutrophils and histiocytes, in contrast to the vehicle group, which predominantly displayed a granulomatous profile in two out of three assays. This finding aligns with the higher abundance of neutrophils (activate and degenerate ones) observed in the epidermis of 80% of the PB8-treated animals.

Histopathological analysis identified a marginal difference in the grading profile for necrosis, with the vehicle group exhibiting higher severity. Although both experimental groups suffered from tissue necrosis, the vehicle group displayed larger patches of necrosis, while the PB8 group had smaller, more focal necrotic areas (data not shown). No other significant differences were observed between these experimental groups.

**Fig. 6:**
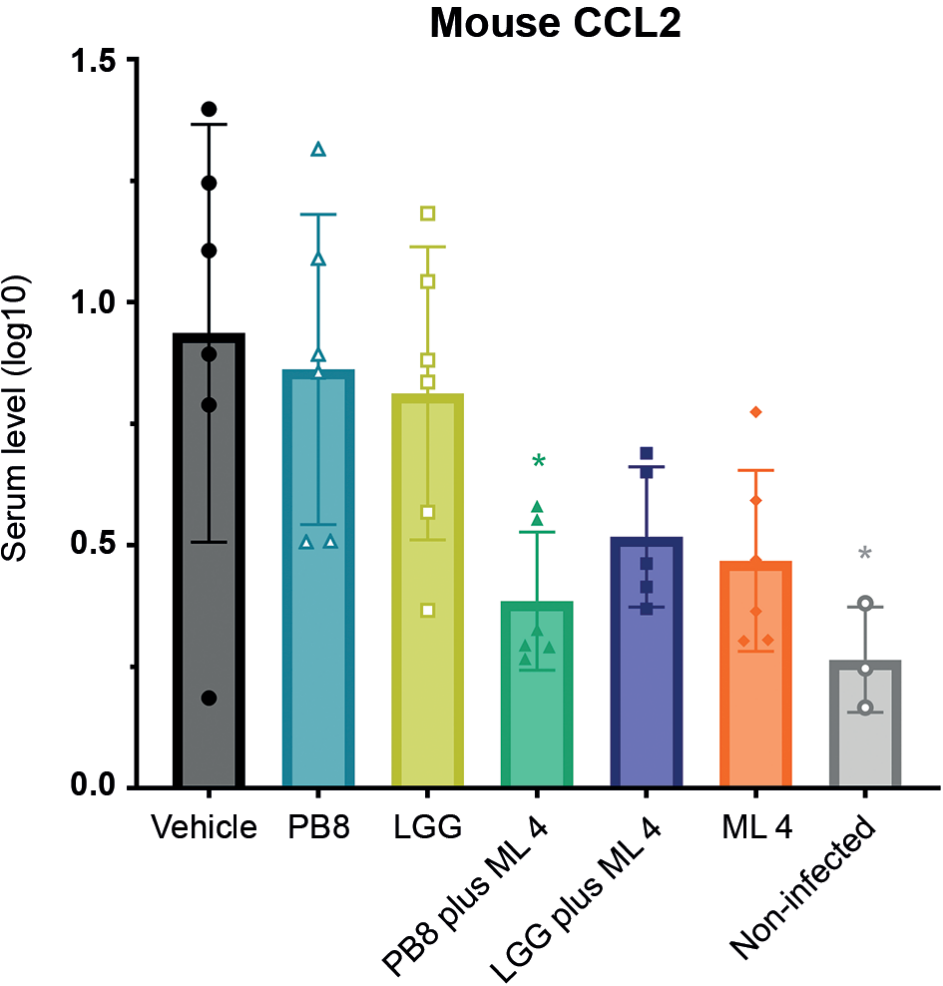
mouse serum monocyte chemoattraction protein-1 (CCL2) levels [pg/mL ± standard deviation (SD)] measured by flow cytometry at the endpoint [50 days post-infection (dpi)]. Male BALB/c mice were experimentally infected with *Leishmania amazonensis* amastigotes and treated according to their respective group assignments (vehicle, PB8 alone, *Lactobacillus rhamnosus* GG (LGG) alone, PB8+ML4, LGG+ML4, ML4 alone and ML40 alone). Three non-infected controls were also included in the assay. Graph design and the statistical analysis [one-way analysis of variance (ANOVA) followed by Tukey’ multiple comparisons test]. Statistical differences relative to the vehicle are indicated by asterisk(s). Levels of significance are denoted as followed: *≤ 0.05.

Inflammatory mediators in mouse sera analysed at the endpoint by flow cytometry demonstrated that *L. amazonensis* experimental infection in male BALB/c mice downregulated pro-inflammatory IL-12p70 and IFN-γ, as well as the anti-inflammatory cytokine IL-10 and upregulated levels of the pro-inflammatory CCL2 and IL-6. The serum levels of TNF were comparable across all experimental groups, including the non-infected and vehicle groups. Statistical analysis showed that only CCL2 was significantly modulated by the combo PB8 plus ML 4 (extended regimen; p = 0.0202) compared to the vehicle-treated group, bringing a range like non-infected *controls* (p = 0.0211) (Fig. 6).

**Fig. 7:**
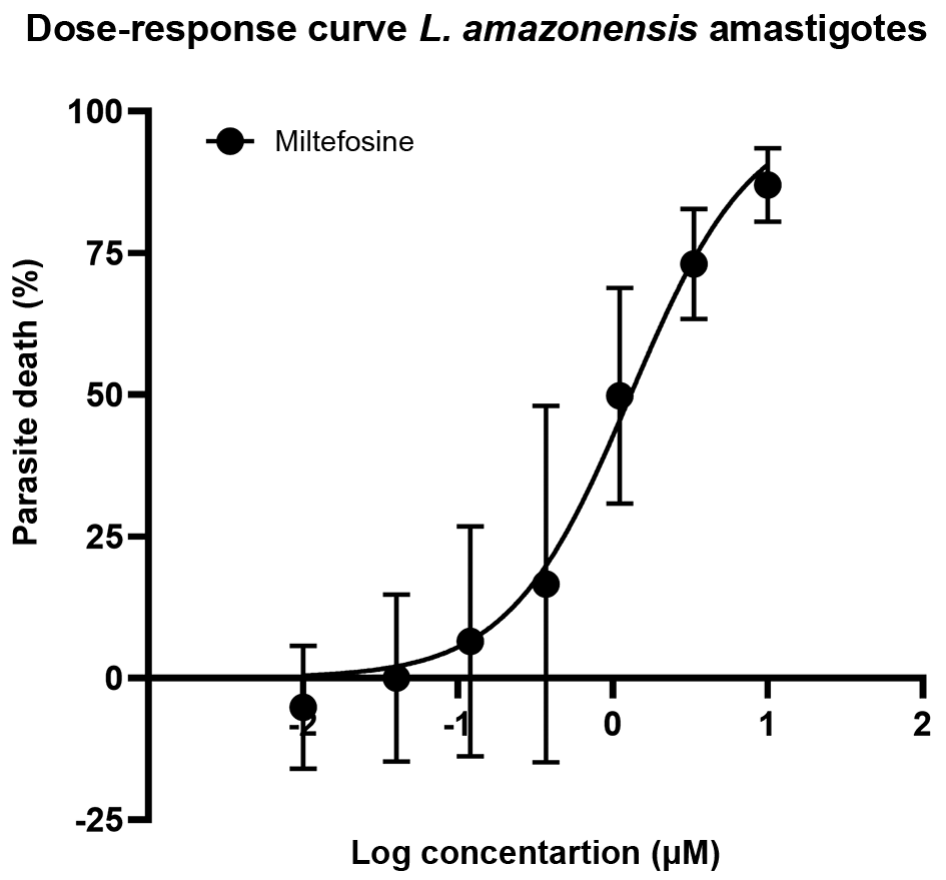
the percentage of dead *Leishmania amazonensis* amastigotes exposed for 48 h at 32ºC to increasing concentration of miltefosine (ML) (10 µM to 0.01 µM). The values represent mean ± standard deviation (SD) replicates of three independent assays. GraphPad Prism v9.3.0 was used to perform the IC_50_ calculation and graph design.

**Fig. 8:**
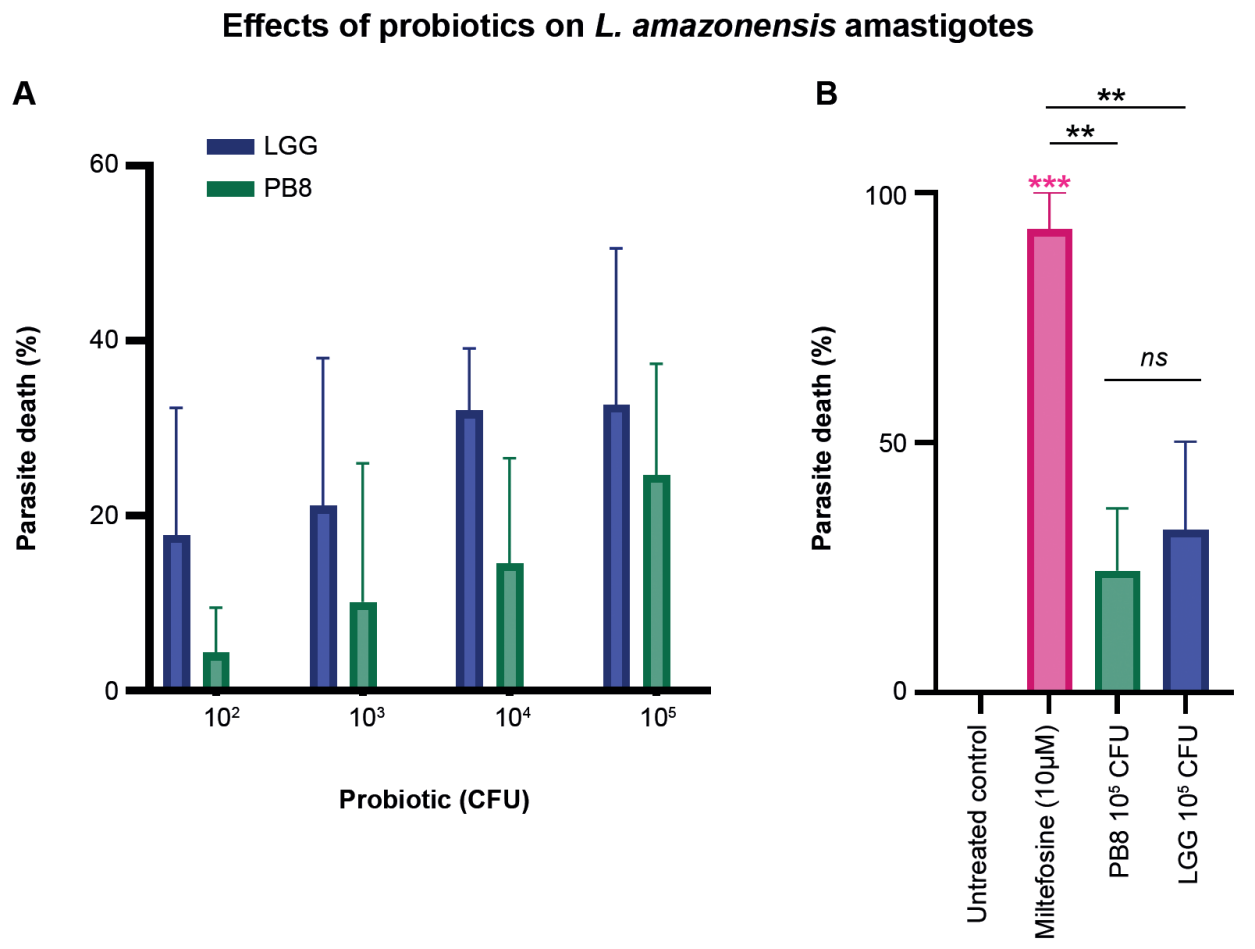
the percentage of dead *Leishmania amazonensis* amastigotes after exposure for 48 h at 32°C to 10⁵-10² colony-forming unit (CFU) of PB8 and *Lactobacillus rhamnosus* GG (LGG) (Fig. 8A) and 10 µM miltefosine (ML) combined to 10⁵ CFU probiotic (Fig. 8B), analysed by light microscopy. Both, an untreated control and ML (10 µM)-treated control were included to this assay. The error bars represent the mean ± standard deviation (SD) of two independent replicates. GraphPad Prism v9.3.0 was used for the statistical analysis [one-way analysis of variance (ANOVA) followed by Tukey’s multiple comparisons test] and graph design. Statistical differences relative to the untreated control are indicated by asterisk(s), while underlined asterisks indicate significant differences between treated groups. Levels of significance are denoted as followed: *≤ 0.05, **≤ 0.01 and ***≤ 0.001.

*Activity against free amastigotes (ex vivo assay)* - In addition to the *in vivo* analysis, we assessed the activity profiles of ML and probiotics towards free *L. amazonensis* amastigotes (Fig. 7). The reference drug ML displayed an IC_50_ value of 1.86 ± 0.09 µM after 48 h of incubation. Regarding the probiotic agents, both PB8 and LGG displayed dose-dependent effects on the free *L. amazonensis* amastigotes after 48 h of incubation, as assessed by light microscopy. At the highest concentration tested (10^5^ CFU), PB8 and LGG achieved maximum parasite death rates of 25% and 33%, respectively, with no statistically significant differences observed between the two across the entire probiotic dose range (Fig. 8). ML treatment (10 µM) resulted in a 93% reduction in parasitaemia reduction compared to the untreated control (p = 0.0003). In contrast, the PB8 (p = 0.0055) and LGG (p = 0.0018)-treated groups induced significantly smaller parasitaemia reductions compared to the ML control.

## DISCUSSION

The World Health Organization (WHO) identifies leishmaniasis as an NTD, characterised by diverse clinical forms. These conditions are attributable to over twenty species of the genus *Leishmania*, transmitted by over ninety sand fly species.^1^ The progression of leishmaniasis is influenced by multiple factors, including malnutrition, inadequate housing, poor sanitation, genetic predispositions of both the host and parasite, environmental and climatic changes, and co-infections.^4^ ^,^ ^12^ ^,^ ^13^

Limited access to healthcare facilities often results in delayed diagnosis of leishmaniasis, which typically occurs after significant disease progression, thereby diminishing the likelihood of successful treatment. The most severe form, visceral leishmaniasis, can be fatal if not treated.^15^ However, the other two clinical forms, CL and MCL, pose substantial challenges. They can lead to ulcers, scarring, disabilities, and stigmatisation with significant social and public health outcomes and are often complicated by secondary infections that exacerbate the disease and prolong patient recovery.^7^ ^,^ ^8^ ^,^ ^9^

As previously stated, conventional treatments for leishmaniasis are outdated and toxic. They encounter increasing issues with parasite drug resistance and high failure rates in endemic regions.^15^ ^,^ ^52^ Furthermore, the mechanisms of action for these drugs still need to be understood. Another concern is that pharmaceutical industries often show limited interest in developing treatments for leishmaniasis due to low financial returns, as the disease mainly affects impoverished populations in low- and middle-income countries.

In terms of prevention and control, antileishmanial methods should be based on vaccination, effective disease surveillance, animal reservoir host control, especially infected dogs and rodents, vector control, *i.e.*, insecticides and bed nets, and detection and treatment of active individual patient cases.^1^ Early diagnosis and effective prompt treatment of active cases are essential for the region and community, as leishmaniasis patients are reservoirs and may cause anthroponotic transmission in endemic areas. In conclusion, the lack of a human vaccine and the high cost and side effects of the current treatments render imperative the development of new therapies that are affordable and easy to administer. Hence, this has stimulated many studies involving the research of new molecules or drug repositioning and new strategies and technologies like cryo-and-heat-based therapies in treating leishmaniasis with potential microbicidal agents and immunomodulators.^52^ ^,^ ^53^

Proposed alternatives for CL include combination or multi-drug uses, drug repurposing, immunomodulators, nanotechnology-based drug delivery systems, and local approaches such as intralesional drug delivery, cryotherapy, and thermotherapy/heat therapy.^8^ ^,^ ^52^ However, most alternative approaches still require further testing before clinical use.

Besides these options, literature has also emphasised the potential of the human microbiota as a potential therapeutic target.^35^ Due to their high activity, stability (longer shelf-life) and low toxicity to humans and other mammalian hosts, probiotics have attracted much attention as potential adjuvant therapeutic agents. However, concerns have been raised about the risks associated with administering live organisms, particularly in vulnerable populations. Possible side effects include virulence factors in probiotic strains, the spread of resistance genes in intestinal bacteria, increased sepsis risk in premature infants, and the formation of persistent colonies that may inhibit normal microflora colonisation.^54^ These risks are particularly relevant for immunocompromised or young individuals, where probiotic use may lead to adverse outcomes.^54^ ^,^ ^55^ Therefore, strict follow-up and monitoring are recommended for their clinical use.

Previous studies from our group observed that probiotic administration in mouse models of *T. cruzi* infection decreases parasitaemia and reduces the *in vitro* infection of peritoneal macrophages when harvested from probiotic-treated animals.^56^ The data corroborates previous reports, which showed that oral and intraperitoneal probiotic administration induces a protective response against the experimental infection with *T. cruzi* in murine models.^44^ Hence, these findings inspired us to study the potential impact of probiotics - multi-strain probiotic blend PB8 and single-strain probiotic LGG - alone or combined with ML against the *L. amazonensis* experimental infection in a mouse model for CL.

In our *in vivo* studies, BALB/c mice were inoculated with the same *L. amazonensis* strain. It is well established that BALB/c mice are highly susceptible to developing characteristic chronic lesions induced by *L. amazonensis*, making them an appropriate experimental model for studying CL.^57^ Our present findings demonstrate that in male BALB/c mice, probiotic daily administration for seven days before *Leishmania* infection, followed by another 14 doses post infection, reduces partially the skin lesion development. At the endpoint, PB8 dropped the lesions sizes up to 32% as compared to the vehicle-treated animals while LGG demonstrated reductions of up to 10%.

Furthermore, histopathology and lesion imprint analysis also demonstrated declines in the average number of amastigotes for both PB8 and LGG monotherapy (20% and 25%, respectively) as compared to the vehicle group. These primary findings suggest that the multi-strain PB8 outperforms the single-strain LGG *in vivo*. Supporting this, several reviews highlight the efficacy and safety of multi-strain probiotic formulations, inducing clinical remission and preventing ulcerative colitis in human IBD patients.^58^ ^,^ ^59^ Similarly, studies have shown that co-culturing of a multi-strain mixture of *Lactobacilli* enhances mucus the binding of *Bifidobacterium (B.) lactis Bb12* and that these synergetic effects could aid in the eradication of pathogens such as *Helicobacter pylori*.^60^ ^,^ ^61^

The gender comparison of PB8 administration in male and female BALB/c mice did not demonstrate statistical differences although previous studies have reported gender differences in experimental *in vivo* models for protozoan infections such as by *T. cruzi*, *L. mexicana*, and *Leishmania Viannia* spp. in male hamsters.^62^ ^,^ ^63^ ^,^ ^64^ As only a single assay was performed using female mice, additional studies are necessary to further evaluate the gender influence on our therapeutic interventions.

In addition to the monotherapy, combination approaches were investigated using the same experimental mouse model for *L. amazonensis*. Combo approaches are largely recommended as it reduces doses and consequently toxic events. ML, an alkylphosphocholine, is the only orally bioavailable drug for leishmaniasis.^16^ ^,^ ^65^ However, it has a narrow therapeutic window, primarily due to gastrointestinal toxicity, including vomiting and diarrhoea, as reported in various clinical trials.^65^ Despite evidence suggesting efficacy against CL, particularly in the Old World, clinical responses to ML are highly variable. Therefore, evaluating multi-therapeutic approaches that include ML is warranted.

ML was administered orally (gavage), starting at the lesion onset (onset = 14 dpi), which ensured successful subcutaneous *L. amazonensis* infection and lesion development. The animals were monitored from 15 to 47-50 dpi to account for the time-dependent effects of ML. Palic et al. state that ML requires metabolic conversion to its secondary active form to exert its therapeutic effects.^16^ Two dosages were assessed: 40 mg/kg/day being optimal for an *in vivo* BALB/c mouse model of CL infection, and a 10-fold lower (suboptimal) dosage of 4 mg/kg/day to assess the potential synergistic or additive effects of combining probiotics with a reduced drug dose compared to the reference drug alone.^66^

Our data indicated that ML 4 monotherapy at a suboptimal dosage at short-term drug intervention (five-day treatment) did not reduce lesion size, as lesions were comparable to those in the vehicle group. Also, the combination with probiotics did not significantly enhance the therapeutic effect of ML 4 alone when given for only five consecutive days. The most significant reduction in lesion size (8.40-fold) was observed in mice treated with high dose ML (40 mg/kg/day) for five consecutive days, supressing lesion development and, consequently, eliminating amastigotes’ nest evaluated by lesion imprints and qPCR analysis.

Interestingly, male BALB/c mice treated with probiotics plus ML 4 but now with a long-term drug administration (twenty-one-day treatment), showed substantially reduced lesion size. The most remarkable reduction was observed in the PB8 plus ML 4 treated groups, resulting in a 74% lesion size reduction compared to the vehicle. In contrast, LGG plus ML 4 and ML 4 monotherapy performed less than their PB8 counterpart, reaching 44% and 53% reduction, respectively. One possible explanation regarding the increased therapeutic efficacy upon extension of the ML administration is its time-dependent activity previously described.^16^ This finding highlights the potential of administering a lower and less toxic dose of ML under an extended treatment regimen in combination with probiotics such as PB8. Given that the standard dosage used in human leishmaniasis often leads to side effects such as gastrointestinal discomfort, this lower dosage could offer a safer compliance.^65^

Presently, the lesion imprint and qPCR analysis support the higher suppression (76% and 87%, respectively) in parasite load parasitaemia induced by the combo PB8 plus ML4, under extended period. It is important to state that since qPCR targets parasite DNA that may derive from both live and unviable parasites and thus, using RT-PCR instead (RNA detection) may improve the methodology to detect only viable parasites as presently quantify in parallel by light microscopy.^67^

Also, continuous administration of probiotics through the drinking water starting after parasite infection was evaluated. However, this approach proved ineffective, as it did not lead to further reduction in lesion size or parasitism as compared to previously studied combination approaches. One possible explanation for this lack of effect could be the difference in administration methods. Probiotics were administered indirectly through drinking water rather than directly via oral gavage. Hence, this raises concerns about whether the specific microorganisms in the probiotic formulation are adequately maintained or stable in the drinking water and whether all mice received a consistent daily dosage of the probiotic. Although drinking water is a more feasible and less stressful method for long-term administration, it has been suggested as less effective than gavage administration.^68^

In addition to quantifying *L. amazonensis* amastigotes, histopathology and imprint analysis by light microscopy were utilised to assess potential differences in the inflammatory profiles between the vehicle and probiotic-treated groups.^51^ Notably, histopathology was performed only during the first *in vivo* assay and was subsequently replaced by imprint analysis, which proved more efficient, less time-consuming and labour-intensive. However, initial histopathological evaluation of epidermal factors (*e.g.*, erosion/ulcers, oedema, hyperkeratosis, and hyperplasia) and dermal factors (*e.g.*, inflammation and necrosis) at the lesion sites revealed no significant differences between the PB8-and vehicle groups regarding absence/presence or grading profile. Interestingly, the vehicle demonstrated a marginally greater severity in necrotic formation at the level of the lesion, and 100% rarefaction in the superficial dermis. These findings indicate that oral PB8 administration could enhance skin quality and support regeneration, like effects reported for other oral probiotics in the skin condition-based literature.^69^

Overall, histopathology and imprint analysis revealed that mice treated with probiotics (10⁹ CFU) alone or with ML 4 exhibited a more pronounced pyogranulomatous inflammation profile than the vehicle group, which generally showed a granulomatous profile (in two out of three independent assays). Granulomatous inflammation is dominated by histiocytes/macrophages (> 50% of cells), while pyogranulomatous inflammation includes both histiocytes/macrophages (30-50%) and neutrophils (50-70%).^51^ ^,^ ^70^ Light microscopy (1000× magnification) highlighted an abundance of neutrophils in the lesions of mice treated with PB8 + ML 4 under extended regimens, suggesting that probiotics may promote an active chronic inflammation compared to controls. Notably, despite prior reports that neutrophil recruitment can worsen lesions in *L. major* infections, none of the probiotic-treated mice in our study showed exacerbated lesions.^24^

Finally, serum levels of IFN-γ, TNF, CCL2, IL-12p70, IL-6, and IL-10 mediator previously implicated in the progression of leishmaniasis were measured using flow cytometry with the Mouse Inflammation CBA kit.^71^ This kit was deliberately chosen to include the main inflammatory cytokines, covering Th1 and Th2 responses, given that the Th1/Th2 balance has been implicated in the clinical outcomes of *Leishmania* infection.^2^ ^,^ ^72^

Our results demonstrated that *L. amazonensis* infection (at the endpoint 47-50 dpi) in male BALB/c mice induced an immunological response characterised by downregulated serum levels of the pro-inflammatory IL-12p70 and IFN-γ, as well as the anti-inflammatory cytokine IL-10 and upregulated levels of the pro-inflammatory CCL2 and IL-6, as observed in two independent assays. Furthermore, differences in TNF levels between the vehicle and non-infected animals were insignificant. This finding is interesting, as TNF has been reported to play a role in the initial host protective (Th1) response during *Leishmania* infection.^72^ However, it is essential to note that serum collection and analysis were conducted at the study’s endpoint (47-50 dpi), indicating that the mice were in a chronic stage of CL infection. Therefore, the observed inflammatory responses may not reflect the robust Th1 responses, typically characterised by elevated levels of IFN-γ, TNF, and IL-12 during the earlier stages of infection, as previously reported in susceptible murine models.^73^

Moreover, the literature suggests that *L. amazonensis* may modulate the host immune system through mechanisms distinct from those of other *Leishmania* species, possibly linked to its ability to infect a broader range of mouse strains.^57^ For example, IFN-γ has been reported to be downregulated in lymph node cells of C57BL/6 mice infected with *L. amazonensis* compared to those infected with *L. braziliensis*.^74^ Similarly, *L. amazonensis* infection in C3H mice resulted in low IL-12 and IFN-γ production levels by CD4+ T cells.^74^ ^,^ ^75^ Furthermore, an *in vitro* study by Belkaid et al. demonstrated macrophages infected with *Leishmania* could not produce IL-12.^74^ ^,^ ^75^ ^,^ ^76^ ^,^ ^77^ These findings are consistent with our observations of reduced IFN-γ and IL-12 levels. Additionally, the reduced presence of lymphocytes at the lesion site following PB8 administration may partially explain the decreased IFN-γ levels, as CD4+ T cells are a major source of IFN-γ.^74^ Furthermore, a clinical study that partially corroborates our findings reported that VL patients unable to clear *L. infantum* infection during drug treatment exhibited decreased levels of protective Th1 cytokines (IL-2, IL-12, TNF-α, and IFN-γ) and increased levels of Th2 cytokines (IL-5, IL-10, and IL-13).^74^ Literature reports indicate that IL-10 plays a crucial role in downregulating the Th1 response in the acute phase of *Leishmania* infection to promote the disease resolution.^57^ ^,^ ^73^ ^,^ ^74^ As we studied the clinical outcome of CL in a later stage of infection (50 dpi), we did not observed significantly elevated IL-10 levels in mouse serum.

Lastly, CCL2 levels displayed notable differences in serum concentrations. The vehicle group had significantly higher CCL2 levels than the non-infected controls, consistent with the current literature indicating that CCL2 plays a crucial role in immunity against CL.^78^ CCL2 mediates the recruitment of macrophages, monocytes, natural killer cells, and other CCR2-expressing leukocytes to the site of infection, which is critical for cellular responses to *Leishmania*, such as increased nitric oxide production enhancing parasite killing by macrophages through CCL2 mediated stimulation.^78^ ^,^ ^79^ Additionally, elevated CCL2 levels have been observed in the skin lesions of *L. major* patients.^80^

In our study, animals treated with PB8 plus ML 4 for an extended regimen showed significantly reduced CCL2 levels compared to the vehicle group, approaching the CCL2 levels seen in non-infected controls. These findings demonstrate that PB8 may modulate CCL2 levels and mitigate the risk of an exacerbated response. It suggests a correlation between reduced CCL2 levels and the observed decreases in these groups’ lesion size and parasitic burden. This finding may appear counterintuitive, as monocytes are generally considered crucial for the containment and clearance of *Leishmania* parasites. However, it is important to consider that *Leishmania* parasites are known to exploit monocytes and macrophages as host cells for replication and survival. Excessive monocyte recruitment could therefore inadvertently support parasite persistence and contribute to chronic inflammation. To illustrate, disease exacerbation in *L. braziliensis* patients has been associated with elevated CCL2 levels, suggesting that this chemokine may contribute to disease worsening by recruiting fresh monocytes to the infection site or influencing downstream events in macrophages and other cells.^81^

In this context, modulating monocyte influx without entirely abolishing it might strike a beneficial balance - limiting the number of permissive host cells available for the parasite while also reducing tissue damage caused by prolonged inflammation.

Additional experiments are needed to confirm the promising effects of the combo PB8 plus ML 4 treatment, which predominantly reversed inflammatory mediator levels compared to the vehicle and non-infected groups, suggesting a potential modulatory effect on parasite control. Regarding TNF, serum levels in mice treated with probiotics, alone or combined with ML 4 (mg/kg/day), were comparable across all groups, including the non-infected and vehicle controls. This finding indicates a minimal impact of the probiotic treatments on TNF regulation.

However, our study is limited by the restricted range of analysed cytokines, which only partially captures the comprehensive biological immune response observed in humans. Specifically, Th17-related cytokines, such as IL-17 - previously reported to be elevated in CL patients, including those infected with *L. amazonensis* ^82^ - were not included in our analysis. Additionally, other inflammatory mediators, often reported to be regulated during *Leishmania* infection, such as IL-4, IL-8, TGF-β, and MIP-1β, could provide deeper insights into the inflammatory profile induced by oral probiotics.^2^ Moreover, collecting serum samples from the experimental mouse groups at different stages, including during probiotic pre-treatment and at multiple time points throughout the infection, could reveal regulatory effects of PB8-administration that were not captured by our endpoint-restricted CBA analyses. Therefore, a promising direction for future research would be to conduct a broader cytokine analysis, incorporating all relevant Th (1/2/17) responses reported for *Leishmania* infection.

We also assessed the direct activity of test agents on axenic *L. amazonensis* amastigotes using AlamarBlue™ spectrophotometry for ML, and by light microscopy analysis for PB8 and LGG. ML achieved IC_50_ value of 1.54 ± 0.86 µM against *L. amazonensis* corroborating previous findings in this experimental animal model.^83^ For probiotic agents, light microscopy quantification showed PB8 and LGG reduced amastigote numbers, with LGG achieving 33% parasite death at 10^5^ CFU compared to 25% for PB8. Higher concentrations were unfeasible due to precipitation and contamination risks. Although the probiotics did not reach 50% efficacy, mild antiparasitic effects from released probiotic-derived substances may directly impact parasitic infection, as suggested in prior studies.^84^ Also, another possibility is that the probiotics may have inhibited amastigote growth in our *in vitro* analysis through nutrient consumption, thereby depleting the culture medium. Both hypotheses need further analysis.

Yuan et al. showed that *Bifidobacterium animalis subsp. lactis BB-12* supernatant significantly impaired *Giardia duodenalis* trophozoite growth and adhesion, causing irregular morphology and growth inhibition.^85^ Similarly, *Lactobacillus* species produce antimicrobial peptides, such as *L. casei* peptides against *Salmonella typhimurium* and *L. acidophilus IBB801’s acidophilin 801*, which inhibits *Escherichia coli* and *Salmonella panama.* ^86^ ^,^ ^87^ Sandipan et al. also found that *Lactobacillus acidophilus*-derived bacterins inhibit *Giardia* adhesion and growth *in vitro*.^88^ Additionally, *L. rhamnosus GG-derived p40* protein has demonstrated immunomodulatory effects in the gut.^21^ Our findings align with these reports, indicating that probiotic-derived metabolites likely contribute to antiparasitic effects. Identifying specific antiparasitic substances by mass spectrometry or chromatography could clarify which strains are responsible, though this was beyond our study’s scope.

In summary, our studies are the first to evaluate the potential benefits of probiotics in experimental mice model of cutaneous leishmaniasis. Our findings demonstrate that a multi-strain probiotic can act as an adjuvant to conventional therapy, enhancing the efficacy of the reference drug ML using lower doses of the reference drug. This was evidenced by significantly reduced lesion sizes and parasitaemia control, as quantified through light microscopy and qPCR. Additionally, we uncovered the immunoregulatory properties of PB8, which modulates CCL2 serum levels, potentially reducing inflammation by limiting monocyte recruitment to the infection site. Further research is needed to optimise probiotic dosing and timing to maximise therapeutic outcomes.

The present results demonstrate that a health gut/skin microbiota (induced by probiotic administration previously given before *L. amazonensis* inoculation) impacts in the lesion development and parasitic load during experimental murine infection. Our present approach aimed to manipulate microbiota composition and functionality to offer host resilience, reducing pathogen colonisation, and fostering a balanced microbial ecosystem that supports immune defence against this parasitic infection. However, another important investigation could be to inspect the potential influence of probiotic administration after the establishment of the parasitic infection, which represents an interesting subject that deserves future studies.

Finally, since the multi strain probiotic mixture currently consists of eight distinct bacterial species and only the LGG strain has been presently investigated, additional studies are desirable to better explore the impact of each separate strain in monotherapy for the observed pharmacological gain. Furthermore, other combinations could be considered, aiming to reduce treatment costs and provide a new therapeutic alternative for this NTD.
